# Controlling Equilibrium Morphologies of Bimetallic Nanostructures Using Thermal Dewetting via Phase-Field Modeling

**DOI:** 10.3390/ma14216697

**Published:** 2021-11-07

**Authors:** Taejin Kwak, Dongchoul Kim

**Affiliations:** School of Mechanical Engineering, Sogang University, Seoul 04107, Korea; xowls4189@sogang.ac.kr

**Keywords:** phase-field simulation, thermal dewetting, bimetallic nanostructure, equilibrium morphology

## Abstract

Herein, we report a computational model for the morphological evolution of bimetallic nanostructures in a thermal dewetting process, with a phase-field framework and superior optical, physical, and chemical properties compared to those of conventional nanostructures. The quantitative analysis of the simulation results revealed nano-cap, nano-ring, and nano-island equilibrium morphologies of the deposited material in thermal dewetting, and the morphologies depended on the gap between the spherical patterns on the substrate, size of the substrate, and deposition thickness. We studied the variations in the equilibrium morphologies of the nanostructures with the changes in the shape of the substrate pattern and the thickness of the deposited material. The method described herein can be used to control the properties of bimetallic nanostructures by altering their equilibrium morphologies using thermal dewetting.

## 1. Introduction

Thermal dewetting on patterned substrates yields metallic nanostructures [1] with a controlled size and arrangement [2] and low cost [3] for various applications, such as high-density recording media and storage devices [4,5,6,7], electron transporting materials [8,9,10], catalysts for the growth of nanowires and nanotubes [11,12,13], phase-change devices using plasmonic nanogaps [14], plasmonic devices for photodetection [15], plasmon-resonance devices [8,16], electrochemical sensing [17,18], and nano-plasmonic polymerase chain reaction (PCR) [19,20]. The properties of nanoparticles, such as dimension, configuration, arrangement, gap, and uniformity, must be predictable and precisely controlled to improve their optical, catalytic, and electronic performances in these applications [21]. For instance, the localized surface plasmon resonance can be tuned by controlling the surface morphology, nanoparticle size, and space between nanoparticles to obtain an optimum surface-enhanced Raman spectroscopy signal from the metal nanostructures at the target wavelength [22]. Additionally, plasmonic hotspots can be intensified using nanostructures with narrow gaps on a fiber-top surface [23].

Thermal dewetting has been used to control and predict the arrangement, spacing, and uniformity of nanoparticles. Although a substrate with separated nano-templates comprising uniformly arranged nanoparticles were fabricated on a flat plate using thermal dewetting [24], additional processes, such as lithographic patterning, reactive ion etching, and wet etching, were required during the process. Gold nanoparticles were previously fabricated on an aluminum dimple array via thermal treatment and the transformation behavior was characterized using thermal dewetting [25]. However, the control of the parameters, such as the gap, arrangement, and size of the nanoparticles, has not been studied yet. Although attempts were made to reduce the gap in the nanostructure, this could only be achieved using an additional thermal dewetting process [26]. The nano-caps and nano-aperture arrays fabricated using a nanopatterned template during thermal dewetting required lithography and allowed only longitudinal control [27]. Although a TiO_2_ nanotube fabricated using thermal dewetting yielded uniform spherical nanoparticles [28], the fabrication of patterned TiO_2_ required lithography and the photocatalytic processing of the Si wafer. The aforementioned studies had relied on the use of diverse complex processes to control the gaps, sizes, and arrangements of nanostructures but had various limitations.

We employed controllable parameters, such as the periodicity and size of the initial pattern of the substrate and deposition thickness, for predicting the arrangement, size, and gap of bimetallic nanostructures in thermal dewetting [21]. We developed a model using the phase-field framework to predict the equilibrium nanostructure morphology during thermal dewetting by incorporating the related mechanisms, including surface energy, interface energy, and diffusion. The current model can be used to quantitatively control the number, gap, and size of the nanostructures, and we developed nanostructures with three equilibrium morphologies (nano-island, nano-ring, and nano-cap) via thermal dewetting and elucidated the mechanism for achieving the equilibrium morphologies with respect to the process conditions.

## 2. Materials and Methods

**Figure 1:**
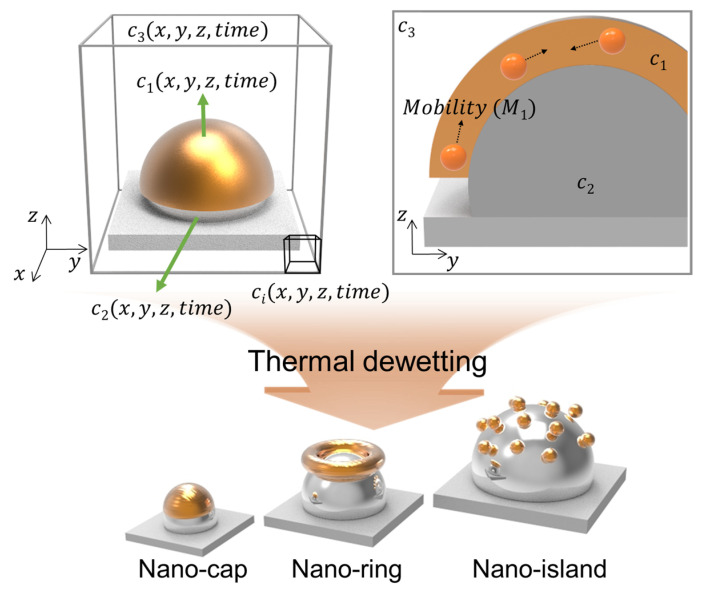
Schematic illustration of the proposed model to predict the equilibrium morphologies and the representative morphologies of a bimetallic nanostructure in thermal dewetting.

Figure 1 presents the schematic illustration of gold deposition along z-direction on a patterned substrate that undergoes thermal dewetting. The three field variables,$\;c_{1}\left(x,\;y,\;z,\;time\right),c_{2}\left(x,\;y,\;z,\;time\right)\;\mathrm{and}\;c_{3}\left(x,\;y,\;z,\;time\right)\;$ represent the volume fraction of the deposited material, substrate particles, and air, respectively. The diffusive flux of the material deposited above the patterned substrate accounts for the diffusion of the material on the substrate during thermal dewetting. The diffusion of the materials induced by chemical potential is expressed as $\nabla \cdot j_{i}$. and $j_{i}=-M_{i}\nabla \mu_{i},$ where $M_{i}$ is the mobility of the material with respect to position. The three-component Cahn–Hilliard model used for calculating free energy (F) and chemical potential ($\mu_{i}=\partial F/\partial c_{i}$) can be expressed as

(1) $$F=\int f\left(c_{1},c_{2},c_{3}\right)+h_{11}\cdot {\left(\nabla c_{1}\right)}^{2}+h_{22}\cdot {\left(\nabla c_{2}\right)}^{2}+h_{33}\cdot {\left(\nabla c_{3}\right)}^{2}+h_{12}\cdot \left(\nabla c_{1}\cdot \nabla c_{2}\right)+h_{13}\cdot \left(\nabla c_{1}\cdot \nabla c_{3}\right)+h_{23}\cdot \left(\nabla c_{2}\cdot \nabla c_{3}\right)dV$$

The local free energy is expressed as

(2) $$f\left(c_{1},c_{2},c_{3}\right)=f_{0}c_{1}^{2}{\left(1-c_{1}\right)}^{2}+f_{0}c_{2}^{2}{\left(1-c_{2}\right)}^{2}+f_{0}c_{3}^{2}{\left(1-c_{3}\right)}^{2}+1$$

The first term on the right-hand side of the Cahn–Hillard Equation (1) represents the volumetric local free energy of the system, and the other terms refer to the interface energies of the patterned substrate and the deposited materials. The gradient energy coefficients are represented using $h_{11},\;h_{22},h_{33},h_{12},\;h_{13},\;h_{23}\;$ and $\;h_{13}$, where $h_{11},\;h_{22}$, and $h_{33}$ are associated with the surface energies of the deposited material, patterned substrate, and air, respectively, while $h_{12},\;h_{23}$ and $h_{13}$ are related to the interface energies between the air, patterned substrate, and deposited material, respectively. The equation for the evolution of bimetallic nanostructures is given by

(3) $$\frac{\partial c_{i}}{\partial t}=\nabla \cdot \left[M_{i}\cdot \nabla \frac{\partial F}{\partial c_{i}}\right]$$

**Figure 2:**
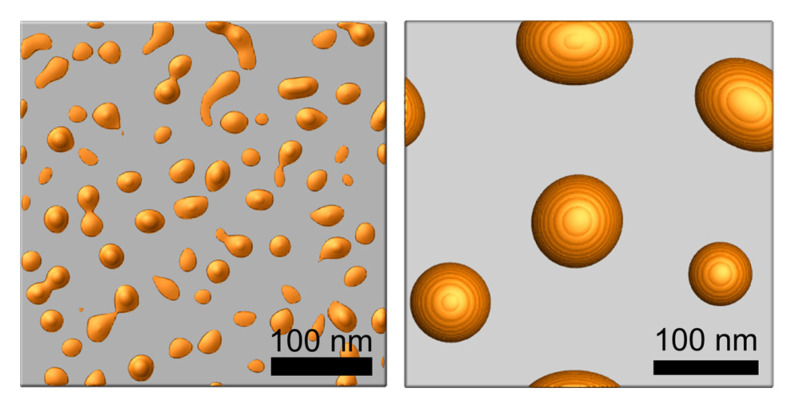
Simulation result of an island-shaped structure y in thermal dewetting with 5 nm (**left**) and 10 nm (**right**) thick Au deposition on a flat substrate.

Simulations were performed with the developed in-house code which calculates the Equation (3) using a semi-implicit Fourier spectral method. Figure 2 shows the island-shaped structures produced via thermal dewetting by depositing 5 and 10 nm thick Au layer on a flat substrate. The average diameters of the island structure (85 nm and 25 nm) and the average gap between the island structures (100 nm and 25 nm) are consistent with the experimental observations [23,29,30].

## 3. Results and Discussion

### 3.1. Effects of the Gap between Patterns in the Substrate and the Deposition Thickness

**Figure 3:**
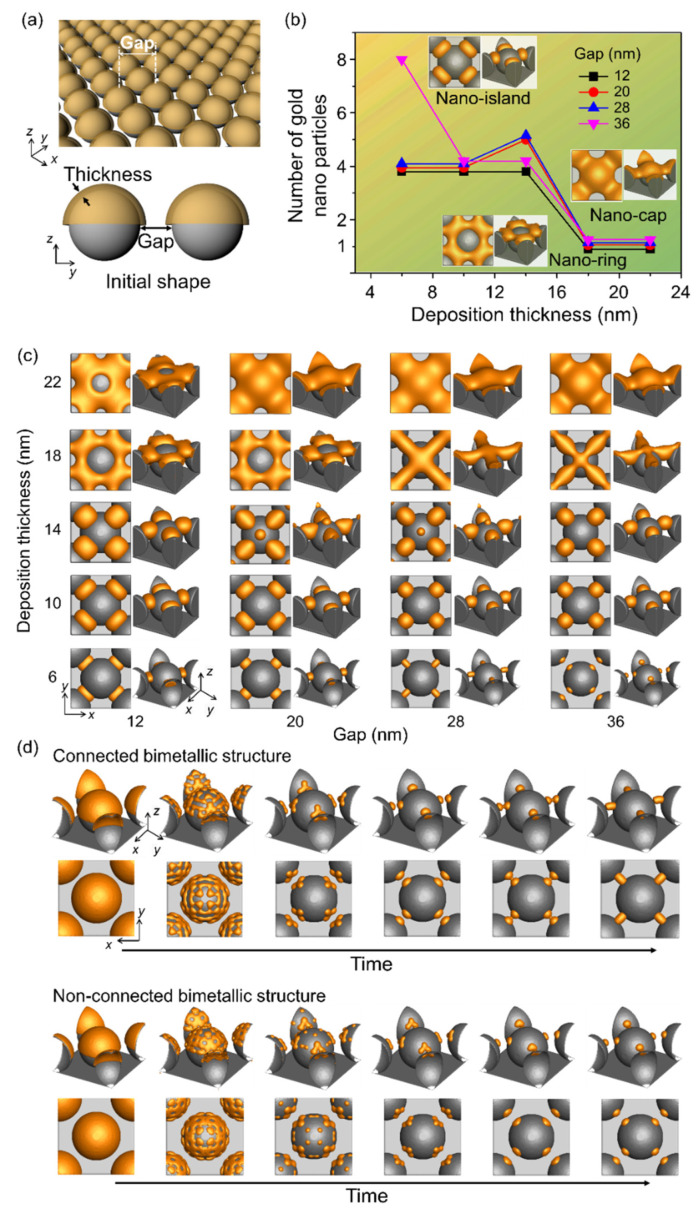
Effects of the gap between the patterned substrate and the deposition thickness on the equilibrium structure in thermal dewetting. (**a**) Schematic of the simulation considering the gap between the patterns in substrate and deposition thickness. (**b**) Number of gold nanoparticles formed by thermal dewetting with varying deposition thickness and gap. The yellow area corresponds to the formation of nano-island, and the green area denotes the absence of nano-ring and nano-cap between the gaps. (**c**) Equilibrium morphologies of bimetallic nanostructures after thermal dewetting with respect to gap and thickness. (**d**) Time-dependent structural evolution of gold nanoparticles that are connected or not connected to the substrate patterns.

We investigated the effects of deposition thickness and gap between the spherical patterns over the substrate on the shape of the bimetallic nanostructures formed during thermal dewetting (Figure 3). Facile fabrication of spherical patterns on the substrate has been reported using nanoparticles and general substrates [31]. Figure 3a shows a schematic diagram of the simulation used to analyze the number of nanoparticles formed during thermal dewetting as a function of the gap between the patterns and the thickness of the deposited materials. When the ratio of the gap to thickness is less than 1 and the thickness is greater than 15 nm, a nano-ring is formed with the deposited material covering the spherical patterns (Figure 3b,c), whereas a nano-cap is obtained when the gap to thickness ratio is between 1 and 2 and the thickness is greater than 15 nm. When the gap to thickness ratio increases beyond 3, a nano-island structure is formed, and an increase in the ratio beyond 6 yields separated structures on the substrate. Particles formed by thermal dewetting are connected if the gap is small, but they are not connected if the gap is sufficiently large (Figure 3d).

### 3.2. Effects of Substrate Pattern Size and Deposition Thickness

**Figure 4:**
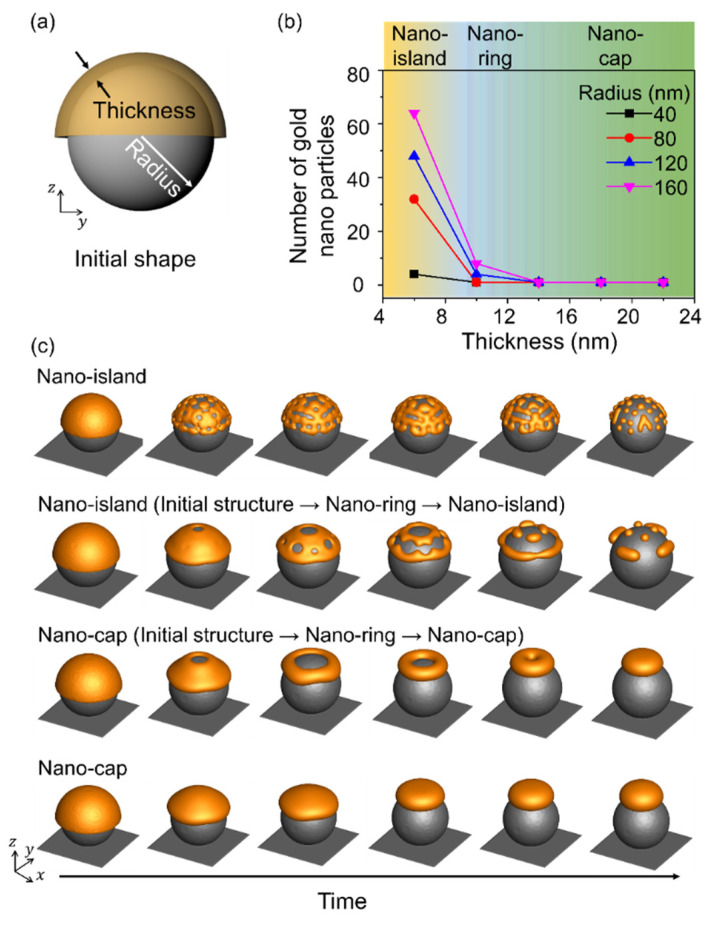
Effects of the radius of the spherical patterns and deposition thickness on the equilibrium morphologies in thermal dewetting. (**a**) Schematic illustration of simulation considering the effects of the radius of the spherical pattern on the substrate and deposition thickness. (**b**) The number of gold nanoparticles formed on the surface of the substrate particle with respect to the deposition thickness and radius. The yellow, blue and green areas correspond to the formation of nano-island, nano-ring, and nano-cap, respectively. (**c**) Time-dependent geometric evolution of the formation of nano-ring, nano-cap, and nano-island.

Figure 4 shows the equilibrium morphologies obtained in thermal dewetting with respect to the size of the substrate patterns and the thickness of the deposited material, and Figure 4a shows a schematic illustration of the simulation. The radius of the spherical pattern corresponds to the size of the substrate pattern, and a sufficient gap was maintained between substrate patterns to distinguish the assembled structures. The nano-island, nano-ring, and nano-cap morphologies were obtained by varying the size of the substrate pattern and the deposition thickness. A radius to thickness ratio less than 6 yielded a nano-cap independent of the thickness of the deposited material and the radius of the substrate pattern. Nano-islands were formed when the ratio of radius to thickness was more than 12, and the number of nanoparticles in the nano-island increased with an increase in the radius to thickness ratio. Nano-rings were obtained when the radius to thickness ratio was in the range 4–16 and the thickness of the deposited material was 10 nm, owing to the retraction at the edge induced by the surface energy of the gold structure [28]. When the radius of the nano-ring increased, it underwent splitting to yield nano-islands, while at a small radius, the structure changed from nano-ring to nano-cap, and the simulation results indicated an increase in the thickness and the radius.

### 3.3. Effects of the Surface Energy of the Deposited Materials

**Figure 5:**
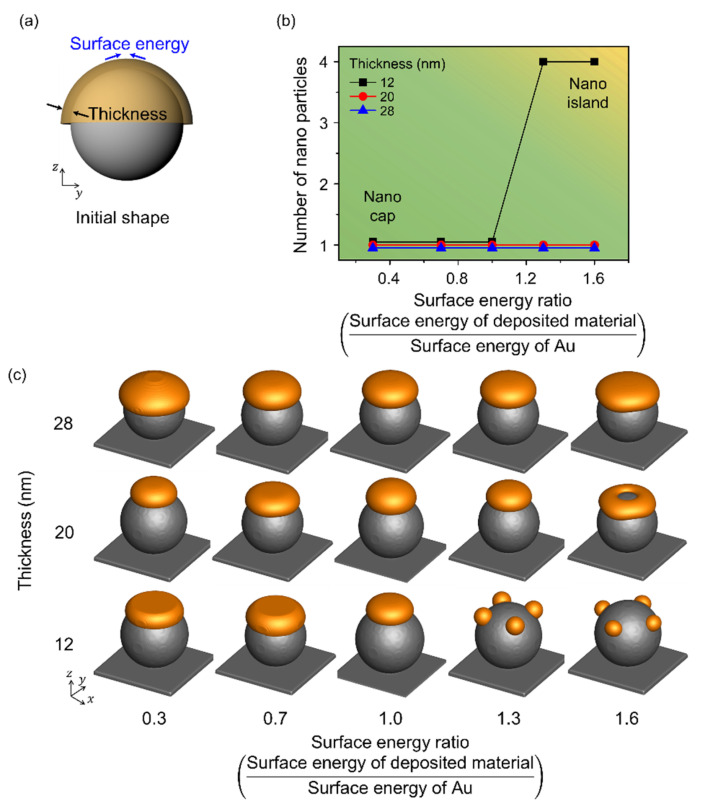
Effects of the surface energy of the deposited material according to thickness in thermal dewetting. (**a**) Schematic illustration of the simulation considering surface energy of the deposited material and deposition thickness. (**b**) Number of Au nanoparticles formed on the surface of the spherical patterns with respect to the change in deposition thickness and surface energy. The yellow and green areas correspond to the formation of nano-islands and nano-caps, respectively. (**c**) Equilibrium morphologies of the nanostructures formed during thermal dewetting with respect to the ratio of surface energy to thickness.

Figure 5 illustrates the change in shape with respect to the surface energy of the deposited material. Figure 5a shows a schematic illustration for the simulation performed considering the surface energy of the deposited material and the deposition thickness, and the radius of the spherical pattern on the substrate was 80 nm. The range of surface energy considered was 0.3–1.6, and it was normalized using the surface energy of Au. When the radius to thickness ratio was less than 4, a single nanoparticle was observed irrespective of the surface energy, which indicated a nano-cap structure. The number of nanoparticles formed by thermal dewetting increased with an increase in the surface energy of the deposited material. When the radius to thickness ratio was more than 7, nano-island structures were obtained. With an increase in surface energy to 1.6, a nano-ring was formed at the deposition thickness of 20 nm due to the increase in the material deposited at the edge resulting in accelerated retraction with an increase in the surface energy. With an increase in the surface energy, the deposited material combined locally, and the number of nanoparticles increased, especially at low deposition thicknesses.

### 3.4. Effects of the Interface Energy between the Spherical Pattern on the Substrate and the Deposited Materials

**Figure 6:**
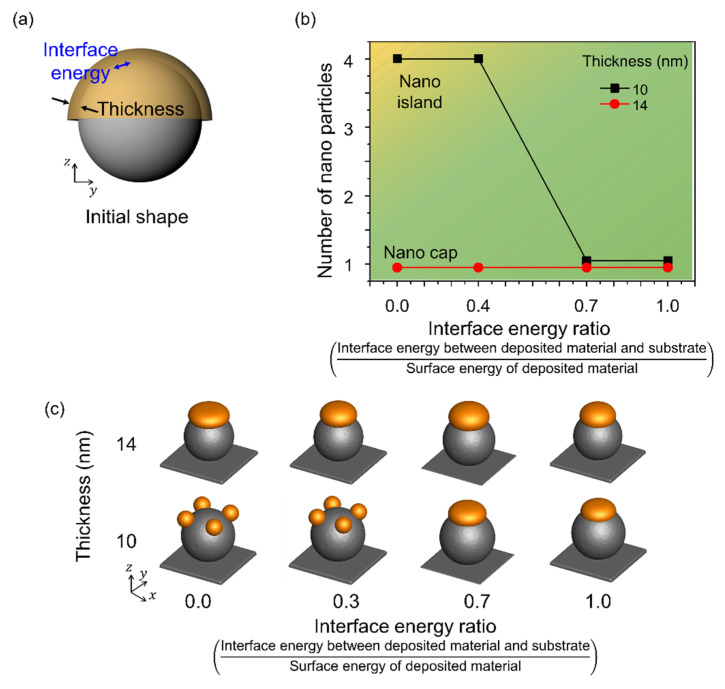
Effects of the interface energy between the spherical patterns and deposited materials. (**a**) Schematic of the simulation considering the interface energy between the deposited material and the spherical pattern and the deposition thickness. (**b**) Number of nanoparticles formed on the surface of the spherical pattern with change in deposition thickness and interface energy. The yellow and green areas correspond to the formation of nano-islands and nano-caps, respectively. (**c**) Equilibrium morphologies of nanostructures formed in thermal dewetting according to the interface energy ratio and thickness.

Figure 6 shows the shape change corresponding to the change in the interface energy of the deposited material. Figure 6a shows a schematic of the simulation performed considering the interface energy between the deposited material and the spherical pattern on the substrate with a radius of 80 nm. The interface energy was considered in the range 0–1.0 and normalized using the surface energy of Au. A single nanoparticle was formed when the radius to thickness ratio was 6 or less, independent of the interface energy. However, the number of nanoparticles formed by thermal dewetting increased with a decrease in the interface energy, and when the radius to thickness ratio was greater than 8, nano-islands were formed. The interface energy increased the contact between the substrate and the deposited material, yielding nano-islands even at low interface energies, and a nano-cap was obtained when the deposited material and the substrate were in contact.

### 3.5. Design of Bimetallic Nanostructures in Thermal Dewetting Process

**Figure 7:**
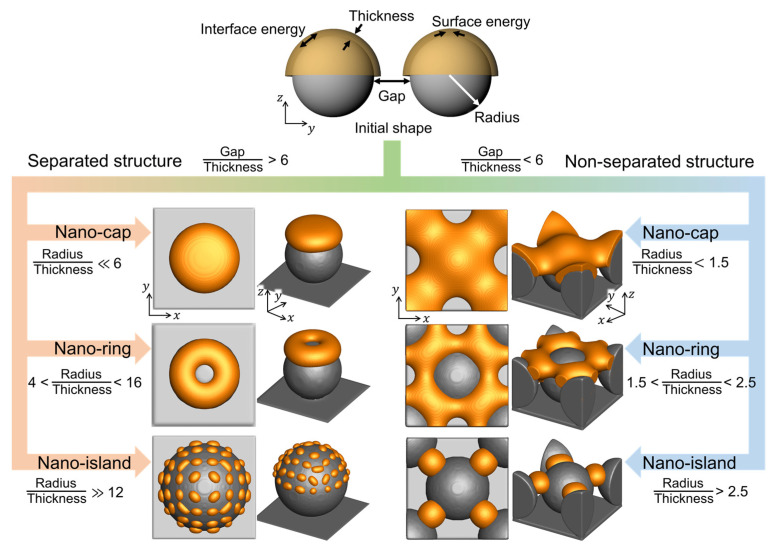
The equilibrium morphologies of nanostructures with respect to the gap, radius, thickness, surface energy, and interface energy.

Figure 7 shows the equilibrium morphologies of the deposited materials in the thermal dewetting process as obtained from the simulation results. The connectivity of the deposited structures was calculated using the ratio of the gap between the substrate patterns to the deposition thickness. The equilibrium morphologies of the deposited materials separated when the ratio reached 6. The equilibrium morphologies, nano-cap, nano-ring, and nano-island, depend on the ratio of the radius of the substrate patterns to the deposition thickness. As the ratio increased, the deposition thickness decreased, and the equilibrium morphology changed from nano-island to nano-cap. With an increase in the amount of deposited material on the same substrate, the equilibrium morphology changed from nano-island to nano-ring, and subsequently, to nano-cap. The predicted quantitative information and the details of the process conditions for thermal dewetting to obtain the desired morphologies are presented in Figure 7. The formation of non-separated nano-islands required a lower radius to thickness ratio than its separated form, and the fabrication of non-separated nano-islands was more straightforward than the separated nano-islands owing to the splitting of the deposited materials caused by the proximity of the substrate patterns.

## 4. Conclusions

We developed a model for the evolution of the bimetallic nanostructures during the thermal dewetting process using a phase-field framework. The developed code with a semi-implicit Fourier spectral method provided simulation results with a high accuracy and efficiency. The quantitative analysis of the simulation results indicates the equilibrium morphologies of the deposited materials as nano-cap, nano-ring, and nano-island. The morphologies of the bimetallic nanostructures in thermal dewetting vary with the change in the thickness of the deposited material and the spacing between the spherical patterns on the substrate. The deposition thickness and the gap between the substrate patterns determine the number and connectivity of the evolved nanostructures. The formation of nano-cap, nano-ring, or nano-island depends on the radius of the spherical pattern on the substrate and the thickness of the deposited material, and the morphologies are obtained at sufficient gap so that the materials deposited on the substrate patterns are not affected by each other. The model elucidates the mechanism for fabricating the equilibrium morphologies of nanostructures with respect to the shape of the substrate pattern and the thickness of the deposited material. This model can be used to fabricate and control the equilibrium morphologies of bimetallic nanostructures through thermal dewetting.

## Data Availability

The data presented in this study are available on request from the corresponding author.
